# GastroTCM: a large language model assistant for gastroenterology in traditional Chinese medicine

**DOI:** 10.1186/s13020-025-01295-8

**Published:** 2026-01-22

**Authors:** Lan Wang, Kaiqiang Tang, Zhichuan Yang, Yan Wang, Peng Zhang, Bowen Wu, Weibo Zhao, Jun Chen, Jiamin Gong, Shiyu Du, Shao Li

**Affiliations:** 1 Institute for TCM-X, MOE Key Laboratory of Bioinformatics, Bioinformatics Division, Department of Automation, BNRIST, Tsinghua University, Beijing, China; 2 Center for Advanced Control and Smart Operations (CACSO), School of Robotics and Automation, Nanjing University, Suzhou, China; 3 Department of Software, Xi’an Jiaotong University, Xi’an, China; 4 Department of Clinical Medicine, China Agricultural University, Beijing, China; 5 Department of Epidemiology and Health Statistics, School of Public Health, Fujian Medical University, Fuzhou, China; 6 Department of Gastroenterology, China-Japan Friendship Hospital, Chaoyang District, Beijing, China

**Keywords:** Large language models, Traditional Chinese medicine, Retrieval-augmented generation, Gastrointestinal diagnostic

## Abstract

Large language models (LLMs) show promise for supporting Traditional Chinese Medicine (TCM) practice, but their clinical utility is limited by domain-specific knowledge gaps, hallucinations, and weak multi-turn reasoning. We present GastroTCM, a specialised LLM assistant for TCM gastroenterology that we built by fine-tuning a Llama3-8B model and augmenting it with a Retrieval-Augmented Generation (RAG) and an agent framework. GastroTCM targets key shortcomings in current TCM diagnostic support through three components: (1) a dedicated TCM gastroenterology vector database for efficient retrieval of high-value, peer-reviewed knowledge; (2) ShareGPT-style multi-turn dialogue optimisation to preserve clinical context across rounds; and (3) an intelligent agent that dynamically adapts its responses to evolving symptom profiles and user intent.

GastroTCM was trained on approximately 20 million tokens of de-identified clinical records, guideline-based content, and expert-curated TCM question–answer pairs and evaluated against strong Chinese LLM baselines (ChatGLM-6B, Qwen-2). In automatic evaluations, GastroTCM outperformed all baselines in single-turn dialogue (BLEU: 0.334 vs. 0.172–0.246) and multi-turn consultations, where it achieved a substantially higher rate of proactive, clinically appropriate interactions (27/60 vs. ≤ 2/60 cases). Expert review by TCM gastroenterologists further confirmed higher diagnostic accuracy and safety, with the RAG module markedly reducing unsupported or hallucinated statements. These findings suggest that domain-specific, retrieval-enhanced LLMs can meaningfully augment—rather than replace—TCM practitioners in gastroenterology, with the potential to improve access to high-quality, explainable decision support in real-world settings.

## Introduction

With the rapid advancement of deep learning technologies, models like GPT and BERT have ushered in the era of large language models (LLMs), which have become a major focus of artificial intelligence (AI) research in recent years. Generative AI chatbots, such as GPT-3.5 and GPT-4.0 [1], harness the power of the Transformer architecture, exhibiting robust language understanding and the ability to perform a wide range of complex linguistic tasks [2, 3]. These models are now being widely applied across various fields, including summarizing literature [4], content generation [5], and predictive modelling [6]. The medical domain stands out as a particularly promising area [7], where LLMs are being leveraged to assist in clinical decision-making, patient communication, and medical research, offering new tools for disease diagnosis and treatment. In gastroenterology specifically, recent systematic reviews have summarized emerging applications of LLMs for endoscopic assessment, cancer risk stratification, and patient-facing education [8, 9]. However, recent evaluations have shown that general-purpose LLMs still make frequent and sometimes clinically important errors when answering medical questions, raising concerns about their safe deployment in real-world care settings [10, 11].

One area of medical focus is traditional Chinese medicine (TCM), which plays a critical role in the prevention and treatment of common digestive system disorders, including chronic gastritis [12]. Research has shown that TCM can aid in gastric mucosal repair, inhibit pathogens such as Helicobacter pylori, and strengthen the immune system. TCM’s gentle nature, with minimal side effects, makes it ideal for the long-term management of chronic conditions [13, 14]. Furthermore, deficiencies in gastric stem cells are a key factor in the development of chronic atrophic gastritis (CAG). Studies indicate that natural compounds derived from TCM can activate gastric stem cells, promoting epithelial regeneration and offering a novel therapeutic pathway for CAG. TCM may also regulate specific signaling pathways associated with gastritis, thereby preventing the progression of gastric cancer. This “inflammation-to-cancer” perspective, which emphasizes personalized and stage-specific interventions, enhances patient prognosis and reduces cancer incidence [15–17]. While Western medicine is known for its precision and standardization, TCM emphasizes holistic care and syndrome differentiation. Clinical experience often shows that integrating TCM with Western medicine can result in superior therapeutic outcomes [18, 19].

Despite the impressive capabilities of LLMs in various tasks, they face significant challenges in specialized fields like medicine. Current LLMs struggle to grasp the complexity and depth of specialized medical concepts, especially when it comes to interpreting intricate medical knowledge. For medical cases requiring nuanced reasoning or contextual understanding, LLMs may fail to fully comprehend the patient’s condition, risking misdiagnosis. Moreover, these models can sometimes generate incorrect or fabricated information, known as "hallucinations," which can mislead users and negatively impact diagnosis and treatment. Recent work further shows that zero-shot LLMs without retrieval or task-specific adaptation tend to hallucinate specialized medical terms due to limited memorization of structured domain knowledge [20]. Studies evaluating the performance of GPT-3.5, GPT-4, and Gemini Pro on medical queries report accuracy rates below 50% [21]. Furthermore, most existing models lack advanced training focused on specific medical domains, particularly TCM. The lack of high-quality TCM datasets further limits the applicability of LLMs in this area. For instance, there is a notable absence of LLM training related to TCM gastroenterology, with minimal research into LLM applications in this domain. These challenges significantly constrain the practical use of LLMs in real-world clinical settings. Addressing these limitations through domain-specific model adaptation and knowledge integration is therefore critical for advancing the use of LLMs in medicine.

To improve LLM performance in specialized domains, researchers have explored various optimization strategies. One prominent method is retrieval-augmented generation (RAG) [22–24], which integrates retrieval mechanisms with generative models. This approach allows for the customization of retrieval strategies and the integration of domain-specific knowledge. For example, Clinfo.ai is a GPT-based RAG implementation that retrieves abstracts from PubMed [24]. RAG not only enhances specialization but also reduces hallucinations and enables dynamic updates to keep pace with rapidly evolving fields. This technique has shown success in numerous clinical applications. For example, the RISE framework, which applies RAG, significantly improved the accuracy and depth of LLM responses to diabetes-related queries [25]. Similarly, retrieval-enhanced GPT-4 has been used to provide clinical recommendations for head and neck cancer patients [26], while a customized GPT-4 Turbo model has demonstrated proficiency in interpreting clinical guidelines for liver diseases [28]. Another key approach is multi-turn dialogue management, which leverages conversational context and user feedback to iteratively refine responses, thereby minimizing hallucinations and enabling accurate, contextually relevant answers. Lastly, vector databases are increasingly used to handle large-scale, heterogeneous, and sometimes multimodal data, thereby enhancing the reasoning and accuracy of LLMs in complex and unstructured data contexts.

The current technologies remain limited when applied to domains like TCM gastroenterology. Existing general-purpose LLMs rarely encode TCM-specific concepts such as syndrome differentiation, pattern–symptom mapping, and classical formula theory, and they lack access to curated TCM gastroenterology corpora. In response to these issues, we propose GastroTCM, a specialized and interactive LLM designed specifically to support TCM-based diagnosis and treatment of gastrointestinal disorders. Built upon the Llama3-8B model, GastroTCM—to the best of our knowledge—is among the first LLMs tailored to TCM gastroenterology, aiming to address the technological gaps in this field. To overcome the challenges associated with understanding TCM’s unique theories and the complex nature of gastrointestinal diseases, we curated and structured clinical data, constructed a TCM gastroenterology knowledge base, and integrated RAG technology with a multi-stage fine-tuning mechanism. GastroTCM functions as a virtual assistant for medical consultations, providing more professional and personalized diagnostic support.

GastroTCM introduces three key innovations. First, it constructs a dedicated TCM gastroenterology vector database to facilitate the efficient retrieval of diagnostic and treatment-related information. Second, it employs multi-turn dialogue optimization (ShareGPT), which enables high-quality contextual understanding and allows for personalized recommendations tailored to users’ specific needs. Third, it integrates intelligent agent functionality, which customizes the RAG process based on real-time symptom descriptions, thus enhancing the model’s applicability in complex diagnostic scenarios.

In this study, we collected extensive TCM diagnostic data and utilized pre-trained models such as GPT-4 for feature extraction to simulate realistic doctor–patient interactions. During training, we applied low-rank adaptation (LoRA) to improve computational efficiency and employed dynamic RAG to enhance the grounding and quality of generated responses in multi-turn dialogues. To assess the robustness of GastroTCM’s, we conducted quantitative evaluations on ten real-world cases, all of which were independently reviewed by certified TCM practitioners. The results demonstrated that GastroTCM consistently provided accurate diagnostic information for TCM gastroenterology, earning endorsement from practitioners.

GastroTCM is not intended to replace medical professionals. Rather, when used appropriately, it is designed to enhance patients’ understanding of their conditions, improve doctor-patient communication, and promote healthcare equity, especially in resource-limited regions. By supporting more tailored, patient-centred recommendations, GastroTCM illustrates the potential role of specialized, retrieval-enhanced LLMs in assisting clinical decision-making in TCM-based gastroenterology.

## Method

**Fig. 1:**
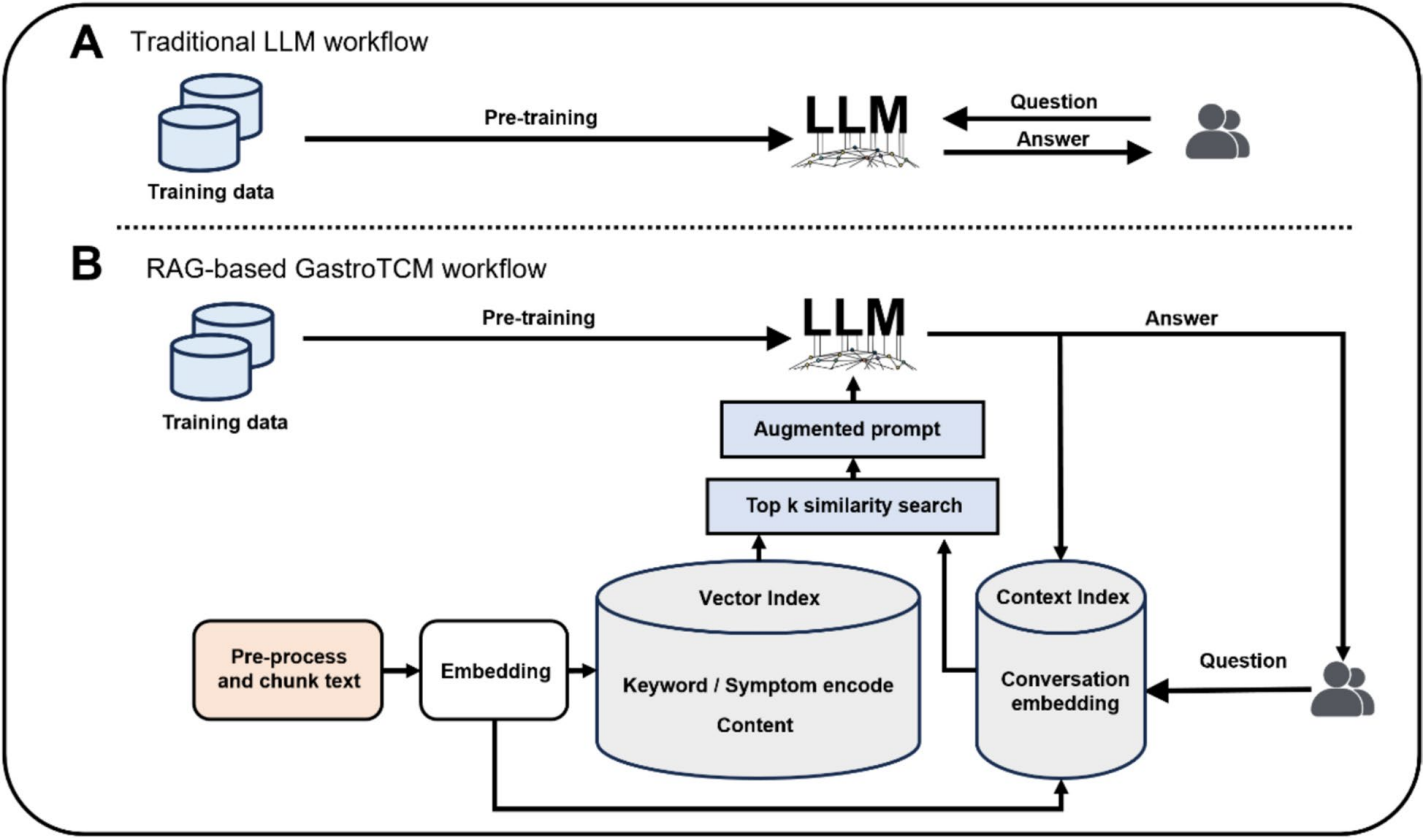
Method overview

We introduce the construction architecture of GastroTCM, as illustrated in Fig. 1, which is built upon the foundation of the Llama3 series of pre-trained LLMs. Developed by Meta, the Llama3 series represents the latest generation of LLMs, having undergone extensive training with diverse datasets to enable both deep language understanding and the generation of diverse textual content.

Among available open-weight large language models, we selected Meta Llama-3-8B-Instruct as the backbone for GastroTCM. Llama-3–based models have been increasingly adopted in biomedical NLP and clinical decision support, and several recent studies have shown that instruction-tuned variants such as BioMed-LLaMa-3 8B, MMedIns-Llama-3, and OpenBioLLM-Llama3-8B/70B achieve state-of-the-art or competitive performance on diverse medical benchmarks. [27] These results suggest that the Llama-3 family provides a strong and data-efficient foundation for building specialised medical assistants. We chose the 8-billion-parameter variant to balance capability with computational cost, enabling on-premise deployment under strict data-governance constraints, while still supporting robust instruction-following and multilingual generation. On top of this backbone, GastroTCM further incorporates a curated TCM gastroenterology vector database and multi-turn dialogue optimisation, which together adapt the model to the TCM clinical context. We acknowledge that benchmarking multiple backbone LLMs (e.g., other open-source models of similar scale) could further improve generalisability and will be an important direction for future work.

The architecture of GastroTCM leverages these advancements to provide a highly specialized, intelligent assistant capable of supporting diverse TCM applications, such as diagnosis, treatment recommendations, and patient management. This combination of Llama3’s core strengths and RAG’s ability to access and integrate external knowledge sources ensures that GastroTCM delivers both depth and precision in TCM contexts, making it a promising tool for practitioners and researchers alike. The overall structure is presented in Fig. 1 for further reference.

### Fine-tuning

Fine-tuning with multi-round clinical dialogs is the cornerstone of our approach because it directly governs robustness and reliability in patient-facing interactions. We curated a Gastro-knowledge QA corpus from internal gastroenterology records (~ 20M tokens) originating from real physician–patient diagnostic encounters. Source materials include de-identified consultation transcripts and free-text clinical notes. We normalized these into ShareGPT-style tuples (system/user/assistant) and further distilled key spans into QA pairs to emphasize symptom elicitation, risk-factor probing, red-flag screening, syndrome (pattern) differentiation in TCM, and guideline-constrained recommendations. For context, our design follows established instruction-tuning practices (Self-Instruct/Alpaca) while adapting them to clinical multi-turn structure.

Because the source data are derived from real clinical encounters and remain privacy-sensitive, we cannot release the raw dataset. Instead, we will release the schema, sampling code, and documentation to facilitate reproducibility without exposing PHI.

To capture clinical conversational dynamics, we constructed 1,000,000 instances, of which 500,000 are explicitly multi-turn dialogs that mimic intake → targeted questioning → differential narrowing → plan/education flows. Dialogs are formatted in ShareGPT style and include system instructions to maintain safety tone and answer scope. We reference public multi-turn resources (e.g., ShareGPT-style collections; medical dialog corpora such as MedDialog) only as formatting precedents; our fine-tuning uses no external PHI.

We use a patient-level 80/10/10 train/validation/test split to avoid leakage, with stratification by subdomain (e.g., gastritis/GERD/IBD/IBS/CAG) and turn-count buckets. Near-duplicate detection (min-hash + lexical overlap) prevents template bleed-through across splits.

To further strengthen domain knowledge, we incorporate ~ 3600 carefully curated TCM-QA instances emphasizing core gastroenterology patterns and classic formula reasoning. These items were authored/checked by TCM clinicians and used for Alpaca-style instruction tuning layered on top of the multi-turn corpus.

**Fig. 2:**
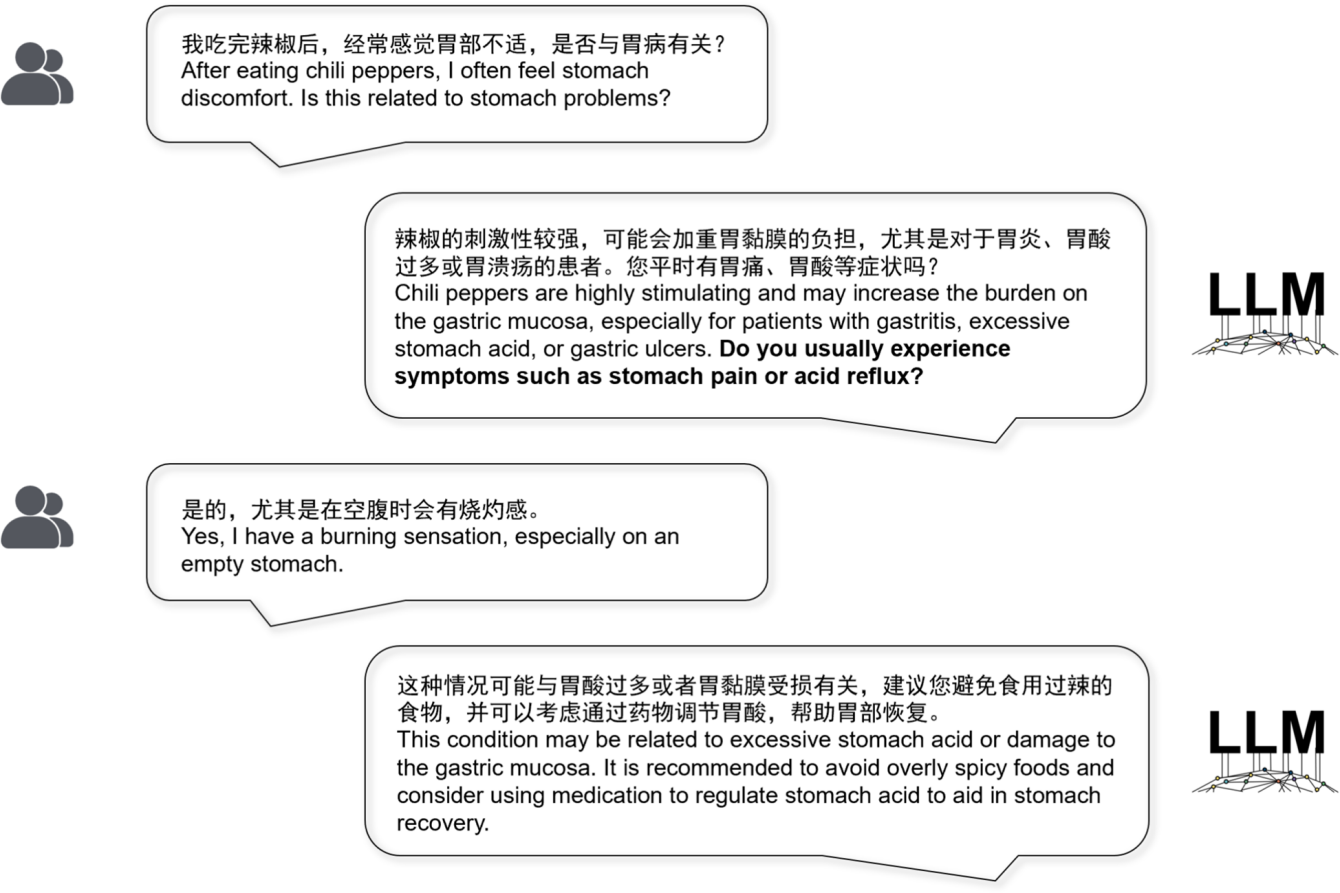
Interactive dialogues overview

In summary, our approach ensures a high degree of professional expertise and interactional accuracy, as shown in Fig. 2, enabling the model to excel in real-world applications within the healthcare domain (Table 1).

**Table 1:** Statistics of the fine-tuning datasets

Name	Content	Instance	Size
Gastro-QA single	Single round patient	0.5 M	408 MB
Gastro-QA multi	Interactive dialogues	0.5 M	123 MB
TCM-QA	Knowledge	3.6 K	10 MB
Total		1,003,600	541 MB

### Model training

**Fig. 3:**
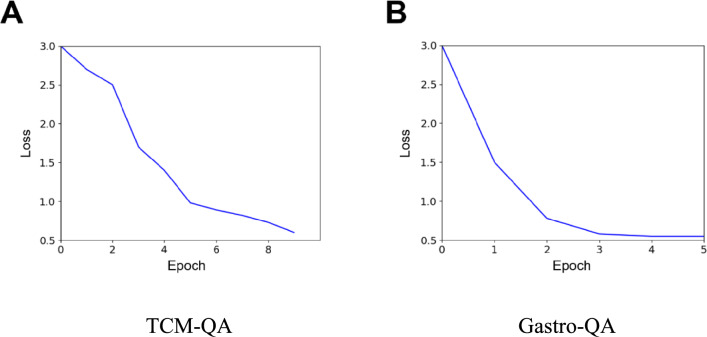
Epoch/loss on Validation

To fine-tune the above model, we utilised a single Nvidia RTX 4090 GPU to ensure efficient and powerful training. The fine-tuning process involved training the model on two specialised datasets: Gastro-QA and TCM-QA [29]. The model was trained for 5 epochs on the Gastro-QA dataset to adapt to the nuances of gastroenterology-related queries, while the TCM-QA dataset, focused on TCM, required a more extensive training of 10 epochs to thoroughly capture domain-specific knowledge. In addition, the LoRA (Low-Rank Adaptation) technique was employed to refine the model’s weights, as shown in Fig. 3, enhancing the model’s adaptability without significantly increasing computational overhead [30]. After completing the fine-tuning process, we merged the parameters of the original model with those of the adapter. This integration strategy allowed us to retain the model’s pre-trained generalization capabilities while effectively incorporating the newly acquired domain-specific knowledge from our private datasets. This approach strikes a balance between maintaining the robustness of the foundational model and achieving high performance on specialized tasks, ensuring the model remains both versatile and highly effective for our specific use cases.

### RAG-agent

Explainability and accuracy are the most crucial in Intelligent Medicine. To enhance the explainability of our model’s response, we apply a RAG-agent framework, as shown in Fig. 1.

To construct a reliable vectorized knowledge database, we utilized the TCM Clinical Diagnosis and Treatment Terminology (GB/T 16751.1-1997) alongside 1,002 TCM research papers. For processing, we divided the text into segments of 2000 tokens each and 1000 overlap tokens and employed advanced language models GPT-4 to identify logical split points within the content. This approach ensured meaningful segmentation of the text into coherent segments.

Subsequently, the extracted segments were further enriched and refined to enhance their clarity and usability when GPT-4 outputs the segments and enhance them with the context, ensuring a structured and comprehensive knowledge representation.

We extracted the keywords and symptoms from every segments using GPT-4. And embedded all segments with keywords or symptoms into distinct items, where each item’s index corresponds to a 512-dimensional encoding vector. The indices of these items are consistently aligned with symptoms or keywords associated with the content. This approach significantly enhances our efficiency and organization.

**Fig. 4:**
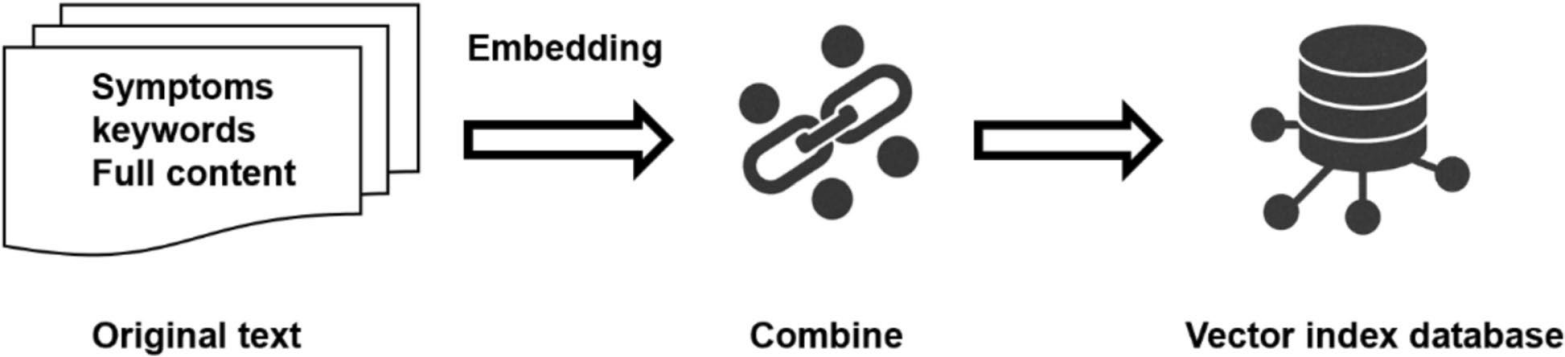
We Embedding the original text to vector and storage into vector index database by keywords\symptoms & full content

After constructing our knowledge database, we match prompts to items during inference (as shown in Fig. 4). As we know, excessive irrelevant RAG content concatenated with the original prompt can significantly reduce the efficiency of LLM inference. Therefore, we use agent-based techniques to filter out some irrelevant context.

1. When we get some results from knowledge databases, we calculate their similarity to the original dialogue and use only the top-k items from the ranked results. Moreover, we will drop the items that do not have keywords in the original dialogues. We refer to this as “Top k similarity search” in Fig. 1.

2. We compare the prompt with the context and calculate its Information Entropy Value (IEV). If the IEV (default 0.5) is below a predefined threshold, we disregard the prompt and take no further action. We called filter 1 in Fig. 1.

This is the IEV calculation formula:

$$IEV=-\sum \mathrm{logcos}(promp{t}_{i},promp{t}_{now})$$

The sum of the logarithmic values of the cosine similarity between the current prompt and the historical prompt and response.

### Evaluation method

To evaluate the precision of our method, we constructed two benchmarks: a single-round benchmark and a multi-round benchmark. Both benchmarks share the same 20 queries in GastroTCM.

The single-round benchmark uses the BLEU (Bilingual Evaluation Understudy) score with the correct keywords we predefined. The BLEU score is a metric used for evaluating the quality of text generated by a machine translation system by comparing it to one or more reference translations. It is based on the precision of n-grams (i.e., sequences of n words) between the machine-generated text and the reference text.

$${P}_{\mathrm{n}}=\frac{\text{number of matching }n-\text{grams in candidate and reference}}{\text{total number of }n-\text{grams in candidates}}$$

For multi-round evaluations, we employ a manual interactive process with the models, where human evaluators engage in multiple exchanges with both the target model and the baseline model. During these interactions, evaluators assess various aspects of the models’ performance, such as relevance, coherence, fluency, and informativeness, based on the context of the ongoing conversation. After each round of interaction, a score is assigned to both the model and the baseline, reflecting their ability to handle the dialogue in a meaningful and contextually appropriate manner.

To ensure a comprehensive evaluation, the following criteria are typically considered:

1. Relevance: How well the model’s responses align with the context of the conversation and the user’s intent. Does the model stay on topic?
2. Coherence: The logical flow of the conversation. Does the model’s response make sense in relation to the previous prompts or responses?
3. Fluency: The grammatical correctness and naturalness of the response. Is the language smooth, or does the model produce awkward or unnatural phrasing?
4. Informativeness: The depth and usefulness of the response. Does the model provide valuable information or offer insights that enhance the conversation?
5. Engagement: The ability of the model to engage in a dynamic, interactive conversation. Does it ask clarifying questions, maintain user interest, or promote further dialogue?

Each model (target model and baseline) is scored based on these dimensions, and the final evaluation score reflects an aggregate of these factors. The scoring can be done on a numerical scale (e.g., 1–5, with 5 being the best) for each criterion, followed by an overall score that summarizes the model’s performance.

## Results and discussion

### Baseline

The benchmark for this study was constructed using dialogues extracted from real-world interactions with 20 gastroenterology patients (GastroTCM Patient BLEU-Benchmark, see Table 2). Keywords relevant to these patients were provided by specialists in the field. To evaluate performance, we calculated BLEU scores by comparing the responses generated by a specialized reference (provided by domain experts) with those produced by the following models: GastroTCM, GastroTCM (No Agent), ChatGLM-6B, and Qwen-2 [31–33]. ChatGLM-6B [34]: ChatGLM-6B is a large-scale pre-trained language model developed by Tsinghua University and Zhipu AI. With 6 billion parameters, it is designed for conversational tasks, offering improved understanding and generation capabilities compared to its predecessors. This model can be applied across various domains for creating more engaging and informative conversational interfaces.

**Table 2:** Problems

ID	Content
1	医生, 我最近吃完饭总是觉得胃不舒服, 感觉食物消化不完全, 偶尔还会有胀气, 是什么原因呢?是不是脾胃虚弱 (Doctor, I’ve been feeling stomach discomfort after meals recently, like the food isn’t fully digesting, and I occasionally have bloating. What could be the cause? Is it due to spleen and stomach weakness?)
2	医生, 我的胃部经常隐隐作痛, 尤其是空腹或者吃得不规律时特别明显。这可能和脾胃功能失调有关吗? (Doctor, I often feel a dull pain in my stomach, especially when I’m hungry or eat irregularly. Could this be related to spleen and stomach dysfunction?)
3	医生, 我的胃肠道有时出现腹泻, 尤其是吃了凉的食物或油腻食物后, 腹泻更为明显。是不是脾胃失调导致的? (Doctor, I sometimes experience diarrhea, especially after eating cold or greasy foods, when the diarrhea becomes more noticeable. Could this be caused by spleen and stomach imbalance?)
4	医生, 我每次吃完饭后肚子都会胀, 感觉非常不舒服, 有时甚至会排气, 感觉像是吃东西后没完全消化。有什么方法能缓解这个症状吗? (Doctor, every time I eat, my stomach feels bloated and very uncomfortable, sometimes even with gas, as if the food isn’t fully digested. Are there any ways to relieve these symptoms?)
5	医生, 我一段时间以来总是便秘, 排便不顺畅, 甚至有时肚子胀气, 感觉很不舒服。我该如何调整饮食或者生活习惯? (Doctor, for some time now, I’ve been experiencing constipation, with difficult bowel movements and sometimes bloating, which feels very uncomfortable. How should I adjust my diet or lifestyle habits?)
6	我吃饭时常常感觉食物很难消化, 胃里总是有种沉重感, 特别是油腻食物后这种感觉更明显。有什么方法可以改善这个问题吗? (I often feel that food is hard to digest when I eat, with a heavy sensation in my stomach, especially after greasy foods. Are there any ways to improve this issue?)
7	每次吃完饭后我都特别困, 感觉没有精神, 想睡觉。是饮食的问题还是身体其他方面出现了问题 (Every time I finish a meal, I feel extremely sleepy, lack energy, and want to sleep. Is this due to my diet or could it be related to other health issues?)
8	我经常感觉到胃里的酸水反上来, 尤其是吃过辛辣或油腻食物后, 感觉很不舒服。有什么办法能解决这个问题? (I often feel acid reflux in my stomach, especially after eating spicy or greasy foods, which feels very uncomfortable. Are there any ways to address this issue?)
9	我的饮食习惯非常不规律, 有时候会吃得很晚, 或者突然吃得很多, 身体感觉越来越不适, 消化也不好。是不是因为这样导致的消化问题? (My eating habits are very irregular; sometimes I eat very late, or suddenly eat a lot, and my body feels increasingly uncomfortable with poor digestion. Could this be causing my digestive issues?)
10	医生, 我吃完某些食物后常常感到不适, 比如水果、乳制品或某些生冷的食物, 可能会引起腹胀、腹痛或者拉肚子。是食物不耐受吗?我应该做哪些检查来确认? (Doctor, I often feel discomfort after eating certain foods, such as fruits, dairy products, or cold/raw foods, which may cause bloating, abdominal pain, or diarrhea. Could this be food intolerance? What tests should I do to confirm?)
11	医生, 我最近压力很大, 特别是情绪紧张时, 胃部就感觉不舒服, 甚至有时会反酸。情绪是不是也会影响胃的健康? (Doctor, I’ve been under a lot of stress recently, and especially when I’m emotionally tense, my stomach feels uncomfortable, sometimes even with acid reflux. Does my emotional state affect my stomach health?)
12	我有时会感觉胃部积气, 打嗝和放屁也比较频繁, 这种情况是不是因为我消化不完全, 还是有其他原因? (I sometimes feel gas buildup in my stomach, with frequent burping and flatulence. Is this due to incomplete digestion, or could there be other reasons?)
13	我有时吃得过多后, 胃部会非常不舒服, 甚至有呕吐的感觉。暴饮暴食对胃有什么影响?怎么才能避免这种情况? (Sometimes after overeating, my stomach feels very uncomfortable, even to the point of feeling like vomiting. What impact does overeating have on the stomach? How can I avoid this situation?)
14	我吃完饭后经常打嗝, 尤其是吃完油腻食物后嗳气特别严重。这是消化不良吗?怎样能减少这种情况 (I often burp after meals, especially with severe belching after greasy foods. Is this due to indigestion? How can I reduce this condition?)
15	我听说食物的加工方式会影响消化效果, 比如吃过多精加工的食物会导致消化不良。您觉得这种说法是真的吗?我应该避免哪些食物? (I’ve heard that the way food is processed can affect digestion, for example, eating too many highly processed foods can lead to indigestion. Do you think this is true? Which foods should I avoid?)
16	我注意到每次吃凉的食物后, 肚子都会胀气, 特别是喝冷饮或者吃冷食时。食物的温度对消化是否有这么大的影响? (I’ve noticed that every time I eat cold foods, my stomach gets bloated, especially when drinking cold beverages or eating cold dishes. Does the temperature of food have such a significant impact on digestion?)
17	我注意到每当我吃得不太好或者吃得很少时, 我的情绪也会变差, 感觉焦虑或者低落。这是因为食物和情绪之间有联系吗? (I’ve noticed that whenever I eat poorly or eat very little, my mood worsens, and I feel anxious or down. Is this because there’s a connection between food and emotions?)
19	我的便便有时很干, 有时很稀, 排便不规律。是不是我的消化系统出了问题?我该如何改善这种情况? (My bowel movements are sometimes very dry and sometimes very loose, with irregular patterns. Is there something wrong with my digestive system? How can I improve this condition?)
20	医生, 我最近吃饭后经常感到胃部灼热, 尤其是吃完辛辣或酸性食物后, 感觉像是胃里在烧。这种情况是怎么回事?有什么方法可以缓解? (Doctor, I often feel a burning sensation in my stomach after eating, especially after spicy or acidic foods, as if my stomach is on fire. What could be causing this? Are there any ways to relieve it?)

Qwen-2 [35]: Qwen-2 is the second iteration of the Qwen series, developed by Alibaba Cloud. This model improves upon its predecessor with enhanced performance, efficiency, and capabilities. Designed for a wide range of applications, including but not limited to text generation, question answering, and conversation, Qwen-2 leverages advancements in machine learning to deliver more accurate and contextually relevant outputs.

### Results

#### Single dialogue

As mentioned above, we evaluate accuracy by comparing model responses with the standard keywords. The BLEU score is shown in Table 2. In order to evaluate more fairly, we restricted candidate tokens to medically relevant terms in this evaluation.

**Fig. 5:**
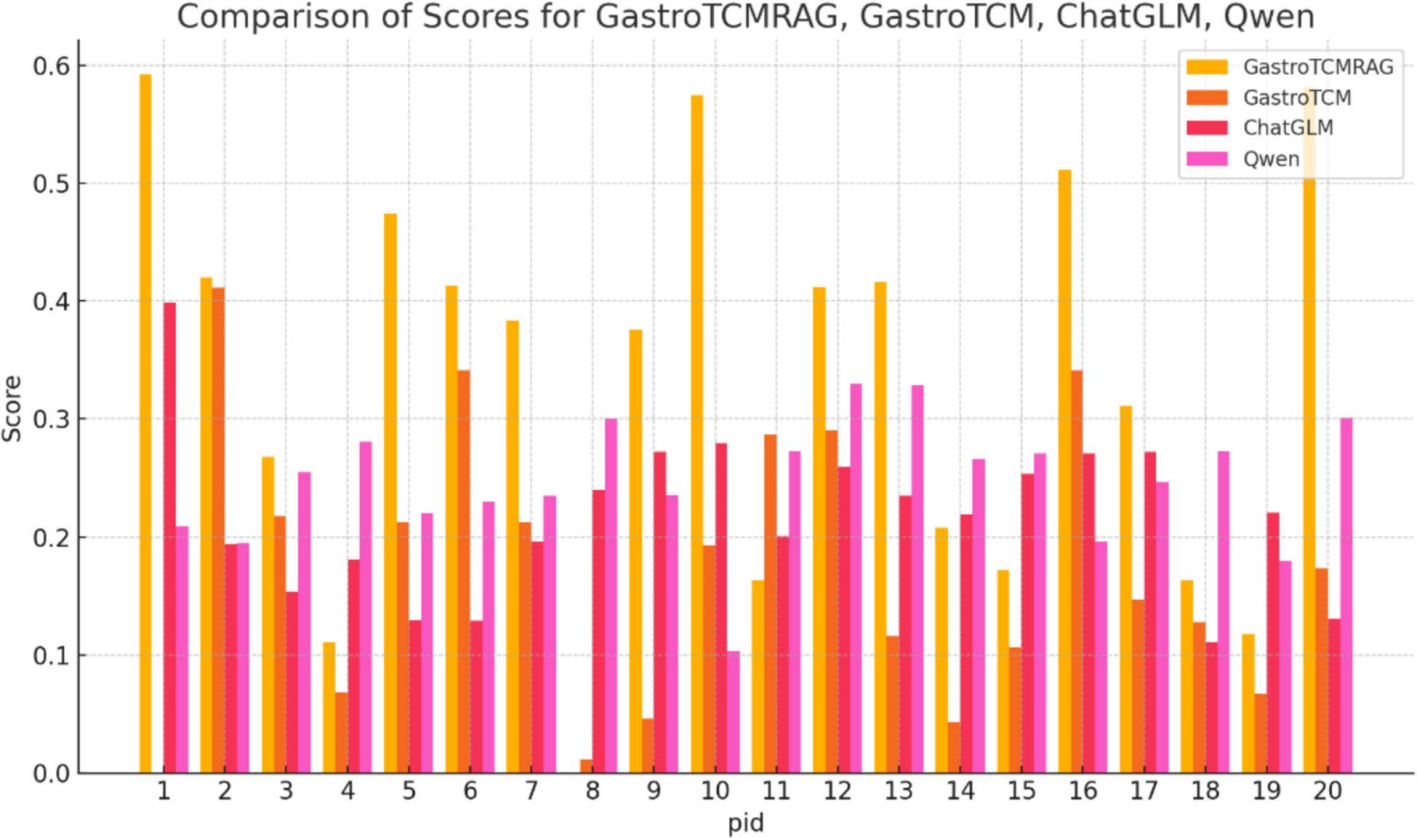
BLEU score per question

After analyzing the results (Table 3, Fig. 5), the RAG-enhanced GastroTCM model significantly outperforms the other models in terms of response quality. However, the Llama3-based fine-tuned models yielded results that were comparable to those of other models, failing to distinguish themselves. This discrepancy can be attributed to the lower Chinese language understanding of the Llama3-based models when compared to alternatives like GLM and Qwen, which demonstrate superior understanding and processing of Chinese.

**Table 3:** GastroTCM Patient BLEU benchmark (BLEU scores)

ID	GastroTCM (RagAgent)	GastroTCM	ChatGLM	Qwen
1	0.592	0.000	0.399	0.209
2	0.420	0.411	0.194	0.195
3	0.268	0.218	0.154	0.255
4	0.111	0.068	0.181	0.281
5	0.474	0.213	0.130	0.220
6	0.413	0.341	0.129	0.230
7	0.383	0.213	0.196	0.235
8	0.000	0.011	0.240	0.300
9	0.376	0.046	0.272	0.236
10	0.574	0.193	0.280	0.103
11	0.163	0.287	0.201	0.273
12	0.412	0.290	0.260	0.330
13	0.416	0.116	0.235	0.329
14	0.208	0.043	0.219	0.266
15	0.172	0.107	0.254	0.271
16	0.511	0.341	0.271	0.196
17	0.311	0.147	0.272	0.247
18	0.163	0.128	0.111	0.273
19	0.118	0.067	0.221	0.180
20	0.581	0.174	0.131	0.301
AVG	0.334	0.172	0.218	0.246

And only the BLUE Score cannot fully capture the overall quality of the response from the models [33]. So we evaluate the TCM LLM conversations in terms of three dimensions: professionalism, safety and fluency. We used “win”, “tie”, and “loss” states as labels, as judged by TCM experts and GPT-4.

The GPT-4 prompt is: Now, you are an expert in Traditional Chinese Medicine (TCM). Please evaluate the following three dialogues from two Large Language Models (LLM A and LLM B). The dialogue from LLM A is enclosed within < A > and < /A > , while the dialogue from LLM B is enclosed within < B > and < /B > . Based on your expertise, compare the quality of responses. If the dialogue from LLM A is significantly better, print ‘WIN’; if LLM B performs better, print ‘LOSS’; if both dialogues are equally good, print ‘TIE’.

The relationship between GPT-4, the experts, and the final result is shown in Table 4.

**Table 4:** The relationship between GPT-4, experts

GPT-4	EXPERT AGENT	RESULT
WIN	WIN	WIN
WIN	TIE	WIN
WIN	LOSS	TIE
TIE	WIN	WIN
TIE	TIE	TIE
TIE	LOSS	LOSS
LOSS	WIN	TIE
LOSS	TIE	LOSS
LOSS	LOSS	LOSS

The result of the experiment is shown in Table 5.

**Table 5:** Win and Loss vs ChatTCM RAG

Name	GastroTCM	ChatGLM	Qwen
ChatTCM RAG loss	0.2666(16/60)	0.4000(24/60)	0.3333(20/60)
ChatTCM RAG win	0.6833(41/60)	0.3333(20/60)	0.3500(21/60)

The RAG-based model again showed the best performance in this setting.

#### Multi-dialogues

Multi-round dialogues, also referred to as interactive conversations, should be evaluated based on their ability to engage in meaningful, back-and-forth conversations. For this evaluation, we used GPT-4 to assess the quality of multi-round interactions. The key criterion for determining the effectiveness of these dialogues is whether the conversation maintains a dynamic flow across at least two rounds, demonstrating the system’s capacity to engage, respond thoughtfully, and build on previous exchanges.

A well-defined interactive dialogue should not only contain a minimum of two rounds but should also foster natural and coherent interactions, maintaining relevance and context throughout the exchange. This evaluation criterion helps judge whether the model successfully simulates a conversational experience or simply provides isolated responses. In our experiments, only GastroTCM proactively asked follow-up questions, whereas the other models did not.

We observed that GastroTCM in Table 6 demonstrates strong performance, largely due to the high-quality supervised fine-tuning data it is trained on. Additionally, the integration of RAG further enhances the accuracy of responses while simultaneously reducing the frequency of proactive questioning [36]. This combination allows for more precise and contextually relevant interactions, ensuring a smoother and more efficient conversation flow.

**Table 6:** Proportion of dialogues with proactive interaction

Name	GastroTCM RAG	GastroTCM	ChatGLM	Qwen
Proportion of proactive interactions	0.4500(27/60)	0.6333(38/60)	0.0166(1/60)	0.0333(2/60)

## Conclusion

This study presents GastroTCM, a large language model (LLM) assistant for TCM gastroenterology, built upon a fine-tuned Llama3-8B model enhanced with RAG technology. Experimental results show that GastroTCM outperforms baseline models in both single-round QA (BLEU score 0.334) and multi-turn interactions (proactive questioning in 27/60 cases, substantially higher than ChatGLM-6B and Qwen-2). Compared with conventional LLMs, GastroTCM’s three core innovations: (1) a dedicated TCM gastroenterology vector database, (2) multi-turn dialogue optimisation (ShareGPT), and (3) dynamic retrieval via intelligent agents, which together improve diagnostic relevance and accuracy while reducing hallucinations (expert-verified win rate of 60%).

Our contributions extend beyond technical implementation to explore optimisation paradigms for domain-specific LLMs such as those for TCM.: First, by integrating RAG with traditional medical knowledge: Structured TCM terminology (GB/T 16751.1-1997) and a repository of 1000 + research papers enable dynamic updating of domain expertise; Second, by fine-tuning on real doctor-patient interaction data (1 million instances) learns symptom-focused questioning patterns that approximate human diagnostic reasoning; Third, by using LoRA technology, training can be completed on a single RTX 4090 GPU, providing a practical pathway for deployment in resource-constrained settings.

Future work will focus on: (1) expanding multimodal inputs (e.g., tongue coating image analysis), (2) improving cross-disease generalization, and (3) conducting real-world clinical trials. GastroTCM is explicitly designed as a physician’s intelligent assistant rather than a replacement, with its core value lying in supporting primary care and contributing to the standardization and globalization of TCM.

## Data Availability

The data of individual deidentified participants will not be shared, but it is available upon request via email: shaoli@mail.tsinghua.edu.cn.

## Appendix

### Training settings

Train type: LoRA.

Learning_rate: 0.000001.

Max generation token: 8192.

Batch size per devices: 2

Gradient accumulation steps: 128.

Num train epochs: 2

Max generation: 2048 for single round and 4096 for multi rounds.

Validation data: 10% of whole data.

## Abbreviations

AI: Artificial intelligence
BLEU: Bilingual evaluation understudy
CAG: Chronic atrophic gastritis
IEV: Information entropy value
LLMs: Large language models
LoRA: Low-rank adaptation
RAG: Retrieval-augmented generation
TCM: Traditional Chinese medicine
